# Medical Transactions of the College of Physicians in London

**Published:** 1821-03-01

**Authors:** 


*Medical Transactions, published by the College of Physicians in London. Volume the Sixth.* 1820.

(Continued from No. III. p. 408.)

In our last Number we gave a full analytical view of the first eight articles in this important volume. Eight more remain to be reviewed, occupying 230 pages of letter-press, and containing a great mass of highly valuable matter. Our analysis, therefore, on the present, and all other similar occasions, must expand in proportion to its freight—and not, like the bed of Procrustes, stretch or restringe it to its own arbitrary dimensions.

ART. IX. *Some Observations on the Nature and Treatment of the Calculous Diathesis.* By WILSON PHILIP, M.D. F.R.S. (Ed.) &c. *Communicated by Dr. Baillie.*

CALCULOUS complaints have, of late years, much occupied the attention of physicians, surgeons, physiologists and chemists. Exclusive *chemical* doctrines and treatment, will not, in all probability, be ever found to apply to any disease. The chemical and mechanical agents, in the human body, are so greatly modified and controlled by the vital powers, as to render all chemical and mechanical explanations unsatisfactory, and the therapeutics growing out of them hazardous. The aid of chemistry, however, is not to be overlooked—and in the class of diseases now under consideration, that interesting branch of science has contributed much to the success of the physician. We agree with Dr. Philip, that Magendie's hypothesis respecting the calculous diathesis is far too chemical. Because azote enters into the composition of lithic acid—because those who swallow much animal food and fermented liquors are liable to calculous disorders—and because animals confined to food containing *no azote*, (and thus having their health and secretions deranged) produced *no lithic acid*, M. Majendie infers that the secretion of this acid depends on the azote received in alimentary substances. Nothing can be more fallacious than such forced experiments; since numerous circumstances must influence the state of the urine, besides the quantity of azote in the ingesta, as Dr. Philip justly observes.

As calculous concretions almost always consist of uric acid, in the commencement of the disease, it is Dr. Philip's object, in the present enquiry, to ascertain, as far as possible, "the circumstances which lead to produce a deposition of this acid in the urinary passages." For this purpose he instituted a series of experiments, twenty-three of which are substantially, though not minutely detailed, and some of which we shall here rapidly glance at.

In the *first* experiment, a young man, in good health, and living on vegeto-animal food, deposited daily from his urine about a grain and a half of lithic acid. But when the addition of three lemons daily was made to the same diet, and under exactly the same circumstances, the amount of uric acid deposition was three grains and a half. The second and third experiments have much the same tendency.

In the fourth experiment, a young man lived wholly on animal food and bread with water, for two days. At the end of this time depositions of the phosphats, but not of lithic acid, were found in different portions of urine set apart on the second day.

After two days' usual mode of living, he ate a lemon on the evening of the latter. On the following day he lived entirely on vegetable substances, except a little broth at dinner, eating this day two lemons. The next day same diet, with three lemons. The urine now deposited lithic acid.

These, and some other experiments of a similar kind, prove, Dr. Philip thinks, that considerable and even slight changes in diet influence the state of the urine. It appears also, from our author's experiments, "that acid and acescent ingesta promote the deposition of lithic acid from the urine, while an opposite diet prevents it, and tends to produce that of the phosphats." But this rule does not hold good always, as the 8th. and 9th. experiments of Dr. P. shew. In the *first* and *fourth* days of the 8th experiment, the quantity of lithic acid was the same, although in the former (1st day) the diet had been wholly of a very acescent nature, consisting of bread, milk, honey, sour cream, and sugar; while in the *latter* (4th day) it was composed of a large proportion of animal food. The 10th experiment shews that the diet may be of the most acescent kind, and even a large quantity of acid taken, without occasioning any deposition of lithic acid. It is also remarkable in this case, that, contrary to Majendie's theory, the increased perspiration, as well as several other causes, lessening the urine, tended to increase the deposition of lithic acid. Still, however, it is true, that, *cæteris paribus,* the less liable is lithic acid to be deposited, the greater the quantity of water in which it is dissolved. Several experiments are related in which the sensible and insensible perspiration was augmented by medicines, in all which cases the urine deposited the phosphats, but little or none of the lithic acid.

"The following difference in the effects of tartrite of antimony and compound powder of ipecacuanha deserves to be noticed. The effect of the latter on the urine is transitory, apparently ceasing as soon as the sweat ceases to flow. That of the former may generally in a greater or less degree, be perceived for several days after it is taken, during which it still seems to lessen the tendency of the urine to deposit lithic acid. I have also repeatedly observed, that the deposition of lithic acid was not so effectually prevented by tartrite of antimony, when it produced nausea, as when no sensible effect was experienced from it, a fact which we shall find explicable on principles we shall presently have occasion to consider." 202.

Dr. Philip observes that we are acquainted with few medicines which more powerfully promote the secretions than mercury; and he has had several opportunities of observing its effects on the urine. The 10th experiment is illustrative of some of these effects. The urine of a young man (a pint in quantity) was ascertained to deposit four grains of lithic acid in the twenty-four hours, without any of the phosphats. He now began to rub in a drachm of strong mercurial ointment daily. In a few days the quantity of urine was reduced to one-half, and deposited no lithic acid, but a copious sediment of the phosphats. In a fortnight the mercury began to disorder the stomach, and general system—now his urine became more copious, and again deposited lithic acid, without phosphats. Dr. P. considers that the diaphoretic action of the mercury, in the first instance, diminished the urine, and prevented the deposition of lithic acid; but that when the stomach became disordered, the skin, from its well-known sympathy with that organ, became less active, hence the increased flow of urine, and the changes in its depositions. These effects our author has observed more than once.

Experiments xxi-ii are brought forward to shew the effects of *indolence*, in increasing the deposition of lithic acid from the urine.

So far back as the year 1786, Mr. Forbes, in a phamphlet on Gravel and Gout, stated that the addition of any acid, even the carbonic, to urine, occasions a deposition of lithic acid. Dr. Philip repeated this experiment, with sulphuric, nitrous, muriatic, citric, and carbonic acids, and always found the result as stated by Mr. Forbes. Dr. P. also found that the addition of an acid prevented the deposition of sediments since discovered to be certain phosphats of the urine. The resumée of our author's experiments may be stated thus:—

"1. That acid and acescent ingesta tend to increase the deposition of lithic acid from the urine, and to prevent that of the phosphats.

"2. That a diet, composed of a large proportion of animal food, tends to lessen the deposition of lithic acid, and to increase that of the phosphats.

"3. That every thing, which promotes the action of the skin, tends to prevent the deposition of lithic acid, and to occasion that of the phosphats.

"4. That dyspepsia tends to increase the deposition of lithic acid and to lessen that of the phosphats, both by producing acidity of the *primæ viæ*, and by rendering the skin inactive.

"5. That indolence has the same tendency, both by inducing dyspepsia, and by lessening the activity of the skin in proportion as it impairs the vigour of the circulation.

"6. That an acid passes by insensible as well as sensible perspiration."

It appears, Dr. Philip observes, from the foregoing experiments, that, however much an acid may prevail in the *primæ viæ*, if the perspiration be free, the deposition of lithic acid is prevented, and a tendency to that of the phosphats produced. "From these facts the inference appears to be unavoidable, that more or less of the acid which, when perspiration is languid, passes by the kidneys, occasioning a deposition of lithic acid, and preventing that of the phosphats, is, when the action of the skin is vigorous, thrown off by this organ." Our author has often observed that it is more effectually thrown off by augmenting the insensible than sensible perspiration—in the former case, he thinks, and with reason, the skin is more active, in the latter more relaxed. Similar observations apply to the kidneys. Dr. P. has reason to believe, that more acid is passed both by the kidneys and skin in the day than in the night. "Even the debilitating effects of nausea seem to lessen the power of the skin to throw off this acid." We may thus, he continues, see how causes, debilitating the digestive organs, may at length give such a disposition to the precipitation of lithic acid from the urine, that this shall take place before it leaves the pelvis of the kidney. The result of Dr. Philip's experiments leave no reason to believe that more lithic acid passes by the kidneys, when we use an *acescent* than a contrary diet. It only appears that in the former case, more than usual of some other acid, capable of precipitating the lithic acid, passes by the urinary apparatus. "Nor have we any reason to believe, when we see the precipitation of lithic acid prevented by diaphoretics, that this acid is thrown off by the skin. We still find it precipitated from the urine on the addition of any *other* acid. But here the diaphoretic has excited the skin to throw off the acid which would otherwise have passed by the kidneys and occasioned its precipitation." If these views be correct, M. Majendie has greatly erred "in directing his attention chiefly to the constituent parts of the lithic acid, and proposing to prevent its formation by preventing the reception into the system of *one* of those parts." It is not the existence of lithic acid in the urine, but its precipitation, that we are called upon to guard against.

The success of the preventive treatment, founded on these principles, is a strong confirmation of their correctness. Dr. Philip has long employed this plan, and, in almost every instance where no concretion had actually taken place, the recurrence of gravel was prevented while the patient conformed to it.

"It consists in such an attention to diet, exercise, and a free action of the bowels as tend to remove and prevent dyspepsia, and to support a due action of the skin, with the use of antacids, of which I have found soap the most effectual." P. 220.

Notwithstanding the disadvantages adverted to by Dr. Marcet respecting the over-use of magnesia, Dr. Philip thinks that, as a preventive of gravel, magnesia, both by correcting acidity in the primæ viæ, and forming a mild cathartic salt there, is a very useful article. Magnesian concretions in the intestines may be obviated by mixing with it a small proportion of finely powdered rhubarb, the taste of which may be concealed by some aromatic.

"It is, I conceive, by removing acid from the primæ viæ, relieving the digestive organs from a load, and rousing them to better action, that *cathartics* are often of great use in obviating the tendency to gravel.'" 223.

Many of M. Majendie's own observations, indeed, shew that dyspepsia is the cause of gravel, and that the means which remove this are the best lithontriptics—at least the best means of avoiding stone. Dr. P. having made many ingenious remarks on Magendie's doctrines, concludes thus:—

"Upon the whole, reviewing all that has been laid before the reader, I cannot help thinking that we have reason to believe, that gravel generally originates in a precipitation of lithic acid in the kidneys, in consequence of a greater than usual quantity of another acid, generated chiefly in the *primæ viæ*, passing by these organs; and that the best plan of prevention is to correct the tendency in the *primæ viæ* to form this acid; to support, by means which invigorate the powers of circulation, the action of the skin, by which in health any superabundance of acid is thrown off; and, when we find that notwithstanding such measures, too much acid still passes by the kidneys, to correct it by antacids before it enters the circulating fluids." 229.

This paper of Dr. Philip's is distinguished by ingenious reasoning and sound principles of practice.

X. *A Contribution towards solving the disputed Question "what is the Nature of the Process called the Spontaneous Evolution of the Fœtus?* By Robert Gooch, M.D. Physician to the Westminster and to the London Lying-in Hospitals, &c.

As an ounce of judgment is worth a pound of learning, so a single fact is worth a dozen of arguments. Without noticing the various disputes respecting the *quomodo* in this very curious Evolution of Nature, we shall proceed to state the case which gave origin to the paper under review.

On Sunday morning, the 24th October, 1819, Dr. Gooch was summoned to a woman, attended by one of the midwives belonging to the Westminster Hospital, and who was in labour with her first child. The midwife reported, that when the membranes had broken, that morning at 4 o'clock, the orifice of the uterus was found somewhat open, but no part of the child could be felt. At length the pains became stronger, and the arm descended, when Dr. Gooch was summoned. When Dr. G. arrived, the arm had not only protruded its whole length, but the shoulder had turned forward under the arch of the pubis, like the occiput just before the head is born. Dr. G. also perceived, that at each pain, the side of the child's thorax was pressed down with great force against the perinæum. The arm, instead of receding, was protruded still farther, and advanced so rapidly, that in two pains, with a good deal of muscular exertion on the part of the mother, the side of the chest, of the abdomen, and of the breech, passed, one after the other, in an enormous sweep over the perinæum, till the nates and legs were completely expelled. The head and other arm were now extricated with the greatest ease. The child had been dead for a day or two before. It was of the full size, and the pelvis of the mother was not particularly capacious. The child being dead, our author thinks, is more favourable to spontaneous evolution, than when it is living. Some positions, too, he thinks, facilitate this process.

"The *most favourable* would be that in which the shoulder is to the arch of the pubis, the head towards the front, and the nates towards the back of the uterus. The *least favourable* would be that in which the shoulder is to the sacrum, the head towards the back, and the nates towards the front of the pelvis."

We have seen only one instance of spontaneous evolution of the fœtus, in the practice of a country surgeon, and where two practitioners had tried, without success, to turn. The arm and shoulder had protruded. We were not present at the moment of expulsion, and therefore, cannot say whether the arm receded before the evolution of the breech; but, the mother died in 12 hours after this dreadful effort.

"If the arm and shoulder," says our author, "protrude far, the pains are strong, and the thorax presses hard on the perinæum, it will be right to wait and watch for a little time; beyond this, whoever suffers a knowledge of this rare fact to discourage him from urning in arm presentations, would be guilty, I think, of criminal irresolution." 247.

This paper is very creditable to its able author.

### XI. *On the Employment of Venesection in Cases of sudden seizures, commonly called Fits.* By JOHN LATHAM, M.D. F.R.S. President of the Royal College of Physicians, &c.

IN several of our late Numbers, we have stated our fears, that ultra-phlebotomy would bring a useful and efficacious measure into disrepute. We have, also, repeatedly exhorted our brethren to practise *local* detractions of blood oftener, and *general* sangui-missions seldomer, than they do. Without going the length which Dr. Latham has here done, in urging objections to blood-letting, on several occasions, we think that his observations are entitled to attentive consideration, and may serve to prevent the improper effusion of human blood, while they are not, we believe, meant to restrain the active employment of the lancet, when the circumstances justly demand the depletory treatment.

Dr. Latham instances hysteria and epilepsy, where the contortions of the patients generally prevent venesection, as doing well enough without it. In cases of syncope, from all the various causes, "the same indiscriminate practice is too often pursued;" and the consequences, in debilitated habits, are often distressing.*

"In apoplexy, although it must be confessed, that blood-letting is seldom so injurious as in those other cases to which I have been alluding, yet, even here, the practice is by much too indiscriminate, and requires, in my judgment, more consideration than is usually bestowed upon it. It certainly ought to make some difference in our practice, whether the patient be of very full or of very slender habit, although the custom of bleeding is religiously observed equally in both, and a fancied *threadiness* or *sharpness* in the pulse is usually considered as a sufficient justification for the immediate loss of blood, notwithstanding the sallowness of complexion, and the flaccidity of muscle may indicate any thing rather than plethora. The lancet however has generally been employed, without any regard either to such circumstances or even to the age of the patient, long before the physician has arrived, and the temporary relief which has been obtained, (for some benefit is commonly experienced in the first instance) is used as an argument for the repetition of the operation, and it is well if by talking about *local* fulness and oppression of the brain

* It must be confessed that the moderns might plead the precept of the ancients, in the sweeping maxim of bleeding every one who falls down in a fit. Celsus seems to except from blood-letting only those cases where convulsions attend. "Ubi concidit aliquis, si nulla nervorum distentio accessit, utique sanguis mitti debet." *Lib.* III. We do not hold this forth as an excuse for indiscriminate blood-letting; but, to shew that, in all ages, men were prone to *routinism.*

he can carry the point so far as to satisfy the bystanders that by leeches to the temples, or by cupping glasses to the neck, a sufficient quantity of blood may be taken more immediately from the neighbourhood of the affected part, than by any depletion from the more general circulation." 252.

Dr. Latham observes, that if a patient of corpulent habit, firm muscles, florid complexion, and a full feeder, be suddenly seized with a fit, corresponding to the usual definition of apoplexy, there can be no question, that large and immediate bleeding from the arm, temporal artery, or jugular vein, together with leeching, cupping, blistering, and other depletory means, would be proper.

"But, in systems directly the reverse, in constitutions flaccid and emaciated, it is surely contrary to sound discretion, that a *similar* mode of treatment should be instituted. It may be, indeed, from oppression on the brain, that the sudden apoplexy has supervened; but we should recollect that congestions may, also, sometimes arise from the *slowness* of the stream, and from the weakness of the impelling force. We should take the trouble of enquiring into the patient's constitution before we proceed to an operation, which, if misapplied, is fatal." 254.

Dr. Latham does not presume to lay down rules that can be applicable to every apoplectic case—"since circumstances must vary and determine the practice in almost every instance." But he thinks that, speaking generally, practitioners called to persons in what are termed fits, should be cautious of the lancet when "the patient is emaciated and the muscles flaccid."

From what has been said, Dr. Latham hopes not to be understood as meaning that, while he condemns indiscriminate bleeding, he is desirous that the physician should stand by and do nothing in such cases. Congestions may exist in the intestinal canal, causing pressure on the larger blood-vessels, and thus producing irregular accumulations of blood in different parts of the system—especially in the head, with all the symptoms of apoplexy. Or gazeous distention of the stomach may produce the same effect. In such circumstances, our author thinks, the prudent physician will evacuate the stomach and bowels (in cases of extreme debility) before he proceeds to empty the blood-vessels. In the mean time, he will have a fairer opportunity of judging of the case, and determining on the propriety of having recourse to local or general bloodletting.

What he has said of apoplexy, he conceives, is generally applicable to paralytic attacks. "The lancet has been employed here too, without due discrimination."

"But, indeed, in these cases, the generality of medical practitioners are not very urgent to employ the lancet, commonly contenting themselves, with what may be termed, a fair depletion by the intestinal canal after one previous bleeding, and then flying at once into the opposite extreme of stimulants and tonics:—a practice which may be characterized as too general to be never wrong, but yet too mechanical to be always proper." 261.

Dr. Latham sums up the object of these observations in the following terms:—

"That venesection is too generally employed, and the relief obtained from it, in the first instances, usually fallacious; that flaccidity of muscles, which depends upon the inanition of the vessels which nourish them, is a better criterion of the actual state of the vascular system, than the pulsation of the artery; and that the unequal distribution of blood, which, even in the weakest constitutions may affect the head, will, in such debilitated habits, be more safely and effectually relieved by other means of depletion, than by *general* bleeding." 262.

Taught by experience and observation, that a *routine* practice is dangerous—especially if of the *ultra* kind, we are advocates for cautious discrimination, in all doubtful cases; while we are the steady friends of energetic practice, wherever the indications point to such measures. Under these impressions, we recommend those who look too exclusively to the vascular system, in their pathology, not to contemn the admonitions of age and experience set forth in the foregoing observations.

XII. *Observations on Puerperal Insanity.* By Robert Gooch, M.D. Physician to the Westminster and City of London Lying-in Hospitals, &c.

This is a very ingenious and interesting paper, and one which we find extremely difficult to analyze. We must take a different course from that pursued by the very able author, in order to render the paper concise, and at the same time perspicuous.

Puerperal insanity attacks a few days, weeks, or even months, *post partum*—nay, it occurs before delivery. Its precursors are generally an increase of frequency in the pulse, sine causa evidente, restless nights, hurry and irritability of temper, wildness and incoherency of language, mania, with not seldom, an attempt at suicide. When it advances in the form of melancholia, it is more gradual, and commonly begins some months after parturition, with, first, a failure of memory, confusion of mind, irresistible and inexplicable depression of spirits, mournful and down-cast countenance, silence and thoughtfulness, self-abhorrence—ending in insanity, which does not differ, in appearance, from the disease in other states than the puerperal. When once begun, its duration is very various—sometimes subsiding in a few days, or even hours; but commonly lasting some weeks or months. In most cases, life is not endangered, nor the intellect ultimately. No fatal case occurred in Dr. Gooch's practice, but some instances have come to his knowledge, in the practice of others. In the Salpetriere, one in fifteen have died of this disease. It is difficult, our author thinks, to distinguish puerperal insanity from phrenitis by the actual symptoms—the history of the case is the best mean of diagnosis.

In phrenetic affections after child-bearing, the patient had, in our author's experience, fever, pain, and throbbing in the head, with, at most, confusion of mind, being always able and willing to describe her feelings. "On the contrary, iu mania, an irritable, incoherent mind was among the first symptoms of the disease."

The ETIOLOGY of puerperal insanity comes within a narrow compass. Almost all we know of it is—the puerperal state—but how or why, we are, as yet, ignorant.

> "Yet there is another cause," says our author, "which greatly contributes to the excitement of this disease—a considerable interruption to that mental tranquillity, so requisite during the susceptibility of the puerperal state—the frequent admission of boisterous persons into the lying-in-room, an officious, eager, irritable nurse, or relative, who, with the best possible intentions, is continually doing the worst possible things—sudden and violent agitation, domestic anxieties, and misfortunes have preceded, and apparently contributed to the appearance of the disease." 281.

Still, as all these things happen repeatedly in parturition, without subsequent insanity, there must be some peculiar predisposition or susceptibility in the brain or nervous system of the individual, called into action by the foregoing exciting causes.

TREATMENT. Our very able author lays down five indications under this head.

1. *Restraint against Suicide.* This can only be properly put in force by a nurse accustomed to the insane.

2. *Removal of Irritation from the Digestive Organs.* As the tongue is usually furred, the alvine excretions unhealthy, and the disease always aggravated, by even a tendency to constipation, purgatives, that will operate copiously twice or thrice, should be given every third day. This class of remedies is superior to every other.

3. *To watch the Circulation, especially the Vascular System of the Brain.* The most difficult question, in the treatment, is, Dr. G. thinks, when to bleed, or not to bleed? The result of our author's experience is, that when there was no evidence of determination to the head, the pulse deviating from its natural state, *only* in rapidity, (probably dependent on mental irritation) blood-letting, general or local, did *not* relieve the symptoms, while it rendered the patient less capable of enduring the exhausting influence of a protracted disease. When, however, the pulse is not only quick, but full and strong, the mental irritation is generally abated by tranquillizing the circulation. When this cannot be effected by *liberal* purging, and cold applications to the head, then a moderate loss of blood from the scalp or neck, by leeching or cupping, will be proper—and still more particularly so, if determination to the head be unequivocally evinced by flushing of the face, redness of the conjunctiva, throbbing and fullness of the carotid branches. Our author, and those, whose opinions he venerates, have come to the conclusion, that general blood-letting is seldom necessary or safe.

4. *To procure Sleep.* The want of sleep is one of the most distressing symptoms both in mania and melancholia. Experience has shewn the inefficacy of opium, even in large doses. The most useful soporific, with which our author is acquainted, is the tepid bath at bed-time, and a night-dose of camphor and hyoscyamus, 10 grains of each. In the Quakers' Lunatic Asylum, meat suppers with porter, are often used as narcotics.

5. *Moral Management of the Mind.* During the violence of the disease, all reasoning is vain. But there is a period when an interview with relations, or such interpositions may be attended with beneficial effects. An interesting case, in elucidation, is here stated, which we shall notice in its place.

We have kept the *didactic*, or preceptive part of the paper uninterrupted by the *narrative*, or illustrative;—a plan which our ingenious author might probably have adopted with advantage. We shall now, however, sketch out some of those cases, which Dr. Gooch has interwoven with the body of the paper.

The first case narrated by our anthor, is one of catalepsy, supervening on puerperal insanity. We shall considerably abbreviate it.

*Case* I. A married lady, 29 years of age, much disposed to hysteria, had had several premature labours, and only one living child. In a few days after her last delivery, in the seventh month of pregnancy, she was seized with violent head and face-ache, which subsided, leaving much flatulence of stomach, with quick, weak pulse, and depression of spirits, soon degenerating into unequivocal and violent insanity, during which she attempted her own life. These symptoms, in a few days, assumed quite another aspect. She was reported by her attendants to be in a trance or dying. Her physicians found, however, that the pupil was obedient to the light. Her eyes were open; but no movement of the chest, nostril, or other indication of respiration, was perceptible. The pulse (120) and temperature alone indicated life. We must regret that our author did not accurately ascertain whether respiration was entirely suspended in this case. That process may be carried on by the diaphragm, without any visible motion of the chest. We do not conceive that the heart would have acted long without its usual and necessary accompaniment, respiration. The lady was now raised so as that the trunk formed an obtuse angle with the limbs—and in that uncomfortable posture she continued many minutes, without support.

"One arm was now raised, then the other, and where they were left, there they remained; it was now a curious sight to see her sitting up in bed, her eyes open, staring lifelessly, her arms outstretched, yet without any visible sign of animation; she was very thin and pallid, and looked like a corpse that had been propped up, and had stiffened in this attitude." 269.

She was now taken out of bed and set on her feet, while loud noises were made in her ears. "But she stood up as inanimate as a statue," although the slightest touch put her off her balance.

She went into this state three several times—the first fit lasting fourteen hours—the second twelve—and the third nine hours; with intervals of one and two days. The disease now resumed the ordinary form of melancholia, and in three months she was well.

Dr. Sutherland related to the author several other cases still more extraordinary—one, in particular, of a young lady who continued cataleptic several months, and was only preserved by great vigilance and management in feeding her. Another patient, a male, being suspected of imposture, was one day placed upright, at the edge of a cold bath, and at length gently pushed in. "He fell to the bottom, like a stone, and continued there without the slightest effort to save himself." This man ultimately recovered. We certainly should, at all times, discountenance these kinds of experiments on real or fictitious cataleptics.

The next case which we shall notice is brought forward by Dr. Gooch in illustration of the gastro-hepatic origin of the disease, and its successful treatment by purgatives.

*Case* II. A lady was seized with puerperal mania, a few days after the delivery of her first child, and which continued long and alarming, but at length she recovered. In her second pregnancy she was attended by Dr. Gooch, in London, and continued well for ten days, when a fire taking place in the neighbourhood, excited the disease a second time. Her nights were sleepless—pulse seldom beyond 100 and soft. "Her face was pale and sallow, and her eye yellowish, her tongue furred, and her bowels scarcely ever acted upon without medicine." Her head was shaved and cupped, without any evident benefit. She took brisk cathartics every second or third day, and in three weeks returned to the country well.

In about a year after this, our author was summoned to attend the third parturition. When Dr. G. saw her she was within a week of her accouchement. She was jaundiced, which the surgeon informed him was the case in her *first* confinement. The tongue was furred—the stools nearly black—the urine high coloured.

"This was a state which it was desirable to correct for its own sake, but still more so when the influence which hepatic disorders have over the mind, even independent of the susceptibily of the puerperal state, was taken into the account, and it was at least a striking coincidence that her former attacks had been preceded by the same appearances. Pills were ordered, each containing one grain of calomel, and five grains of the myrrh and aloetic pill, and she was ordered to take one often enough to move the bowels three times daily. One a day was sufficient—her tongue became clean—she lost the yellowness of her eye and complexion—the stools assumed a natural appearance, and she fell in labour about nine days afterwards, with her stomach and bowels in a natural condition; her friends, and the attendants who had witnessed her former illness, were long before they lost their apprehensions; but this time she passed through her confinement without the slightest appearance of her former disease." 287.

Dr. Gooch ingenuously confesses that this case is not likely to make so great an impression on others as on himself; and that he can excuse them for doubting whether the treatment was the cause of the escape. In the following case, however, there can be little question that the remedies employed were the cause of the striking relief experienced.

*Case* III. A lady, literary, talented, susceptible, was anxious to nurse her first child; but her nurse and surgeon thought she could not. Irritating discussions ensued, and puerperal mania was the result. Her skin was hot, her pulse was full, and above 100, her tongue darkly furred, her bowels confined, her stools excessively offensive and dark. A dose of calomel and jalap, with salts, procured a few evacuations, but produced little relief. She was therefore ordered still more active cathartics, which expelled a very large evacution, nearly black, and horribly offensive.

"This was as usual discharged into the bed, without any notice on the part of the patient. It acted again an hour or two afterwards; but now the nurse, who was sitting by the bed-side, was surprised to see her turn round, and in a calm and natural manner request to be taken up as her medicine was going to operate; her waistcoat was immediately loosened, and she was taken out of bed, when she voided a stool of prodigious size, as dark and offensive as the first, and then walked back to her bed calm and collected." 290.

The physicians saw her a few hours afterwards lying on the sofa, perfectly tranquil, answering questions correctly, and manifesting no vestige of her former complaint, excepting some little strangeness of expression in her countenance, and a timidity in conversation. Her recovery was rapid and perfect.

The influence of the biliary organ on the mental functions was noticed by philosophers, physicians, and even poets, among the ancients; several of the moderns have also stated facts in support of this connexion. Within these few months M. Bricheteau, an able pathologist, has published a memoir on this very subject, an analysis of which will be found in another part of this journal.*

The next case which we shall abstract is one to illustrate the beneficial effect of moral emotions on the alienated mind, at a certain period of the disorder.

*Case* IV. A lady, ætat. 28, of susceptible mind, but good constitution, became affected with melancholia a few months after her second accouchment, owing apparently to a frightful incident that had occurred towards the end of her pregnancy. Having weaned her child, gone to the sea side, and taken some tonic medicine, without benefit, her spirits became gradually more depressed, and nothing could persuade her that she had not some fatal disease.

---

* Quelques considerations de physiologie pathologique sur les rapports particuliers et reciproques qui existent entre le cerveau et le foie. Par M. Bricheteau. *Journal Complementaire, Sept.* 1820.

"One day it was cancer, another inflammation of the bowels: and to such a height did her apprehensions rise, that her husband was often brought home by some alarming message, and found her, with a solemn air, and in a low whisper, giving directions to her servants whom she had assembled round her, what to say if she should expire before their master arrived." 295.

It was now evident that she was deranged. She was sent into the country, under proper attendance, and separated from husband, children, and friends. For several weeks she manifested no improvement, sometimes occupied with one notion, sometimes with another. At length one predominant hallucination absorbed all the rest. She was doomed to a public and disgraceful execution for her crimes—every noise she heard was that of the workmen preparing the scaffold—every carriage was that of the officers of justice assembling at the place of execution. But the worst of all was the disgrace and death of her children and husband, whose spirit haunted her. A white post at the back of the cottage was her husband's ghost, which, day and night, was whistling in her ears. Several weeks passed this way; but her husband at length determined on an interview. The scene which passed at this interview we shall give in the words of the husband.

"As soon as I entered the drawing room, she ran into a corner, hid her face with an handkerchief, then turned round, looked me in the face, one moment appearing delighted at the thought that I was alive, but immediately afterwards assuming a hideous expression of countenance, and screaming out that I was dead and come to haunt her.

"Finding that persuasion and argument only irritated and confirmed her in her belief, I desisted, and tried to draw off her attention to other subjects; it was some time since she had seen either me or her children; I put her arm under mine, took her into the garden, and began to relate what had occurred to me and them since we parted; this excited her attention, she soon became interested, and I entered with the utmost minuteness and circumstantiality into the affairs of the nursery, her home, and her friends. I now felt that I was gaining ground, and when I thought I had complete possesion of her mind, I ventured to ask her in a joking manner whether I was not very communicative for a ghost; she laughed; I immediately drew her from the subject, and again engaged her attention with her children and friends. The plan succeeded beyond my hope; I dined, spent the evening with her, and left her at night perfectly herself again." 300.

She had no relapse. Dr. Gooch does not mean to draw the conclusion from this case that violent mania is curable by conversation, but that there is a stage approaching convalescence, in which the bodily disease is loosening its hold over the mental faculties, and in which the latter are capable of being drawn out of the former by judicious appeals to the mind.

The remainder of Dr. Gooch's paper is taken up with an inquiry into "the grounds of the *prevailing doctrine* that mental derangement is a moral disease." We certainly were a little surprised to find Dr. Gooch stating the above to be a *prevailing* doctrine; for we believe that hardly a *pathologist* of the present day entertains any such opinion. This the readers of this journal need not be told; since hardly a number has issued from the press without containing the sentiments of enlightened authors on the subject, diametrically opposite to what Dr. G. supposes to be the prevailing doctrine.*

That the intellectual principle within us should be subject to disease like a liver or stomach, is quite absurd, whether we look upon that principle as an immortal spirit, or a mere function of the brain. To the materialist we need not address our observations, for he can have no difficulty in conceiving insanity to be a corporeal disease; but the Christian and Platonic philosopher seeing purely *moral* causes produce aberrations of the *intellect*, is naturally led to enquire how this can be a *corporeal* derangement. In the first place, we are to recollect that all the operations of mind are revealed *through* matter; although this revelation does not prove the identity of mind and matter. Does music prove the flute and the performer to be the same thing? We cannot possibly have music without an instrument, whether that instrument be an artificial contrivance of wood or wires—or a natural apparatus, as the larynx. It is so with the manifestations of the human soul. It must have a brain to manifest itself through. And as, in the case of the musician, a broken string or an obstructed tube will derange the operations of a Handel; so a corporeal disease of the brain, whether idiopathic in that organ, or symptomatic of other affections in the system, must inevitably derange the intellectual operations.

But if corporeal disease be the cause of insanity it does not follow that the corporeal disease itself results from material causes. A man takes up a newspaper and reads a frightful piece of intelligence respecting some of his family. The process of digestion in the stomach is instantaneously arrested, and his tongue becomes dry and coated in a few minutes. Or an officer is publickly insulted, and, in the vio-

* See for instance, the Reviews of Esquirol, Haslam, Burrows. &c. &c.

lence of his rage, becomes quickly *jaundiced,* delirious, and dies.* Now if a moral impression on the sensorium be capable of instantly disordering the functions of various distant organs, is it not likely to suffer in its own structure or functions, both by these primary impressions, and through consent with those other organs secondarily affected? Undoubtedly it is; and it is in this way *principally* that the sensorium becomes deranged either in structure or functions, and consequently incapable of manifesting correctly the operarations of the mind or soul—in other words, insanity is *generally* produced by moral causes inducing corporeal disorder, and that corporeal disorder embarrassing the intellectual functions.

This view of the etiology and pathology of insanity leads, we believe, to the most successful treatment. As moral and physical causes combine in the production of the disease, so moral and physical remedies must be combined in the removal of it.

> " Sunt verba et voces quibus hunc lenire dolorem
> " Possis, et magnam morbi depellere partem." *Hor.*

The above, we believe, are the sentiments of the best modern physicians, and they are those which Dr. Gooch has in this paper laboured to maintain. No one can read the article in question, without being impressed with a deep conviction that its author possesses talents of a very high order, brightened and improved by careful observation and mature reflection.

XIII. *Some Observations on the Duodenum, with plates, descriptive of its situation and connexions. Extracted from the Gulstonian Lectures.* By G.D. Yeats, M.D. F.R.S. Fellow of the Royal College of Physicians, &c. &c.

Very little doubt can be entertained that the office of the duodenum is nearly, if not quite, as important as that of the stomach; and when we consider that it receives the secretions of two other organs (the liver and pancreas) one of which is liable to numerous derangements, there can be no question that this second stomach is the seat of a variety of ailments,

* " Un jeune militaire recoit un soufflet dans un lieu publique, et dans la fureur qui le transporte, il tire son epée pour percer celui qui vient de lui faire une insulte aussi grave. Presque au même instant, il devient icterique, et bientôt après il est pris de fievre avec delire, et meurt au milieu de convulsions." *Bricheteau.*

both of function and structure, though not noticed in any of our modern systems of practical medicine. "Et quemadmodum usus amplissimus (says Hoffman, de duodeno, vol. iv. page 190) et longe maximus est illius; ita etiam si vitium vel defectus in illud incidit, si intemperie menstruales succi laborant, si debitus tonus hujus eximiæ partis læsus vel destructus fuerit; magna inde turbatio in universa corporis œconomia et ingens malorum proventus exsurgit."

As this is a very important subject, and Dr. Yeats's paper a very able one altogether, we shall be excused for giving not only a minute analysis of it, but for collecting observations that bear on this point of pathology from various other sources.

Dr. Yeats very properly opens this dissertation with some remarks on the physiology of the duodenal digestion. The pultaceous mass is passed from the stomach into the duodenum, with its solid parts in a diminished state of aggregation. It now becomes mixed with the secretions from innumerable glands, more particularly situated at the commencement of the duodenum. The chyme is thence propelled to the sacculated angle of the gut, where it is attached to the capsule of the right kidney. Here it receives the hepatic and pancreatic secretions, and undergoes a change by which is produced chyle. Dr. Yeats is inclined to attach some importance to the connexion of the duodenum with the colon, whereby the motions of the one are communicated to the other.

"In this way it is probable a great part of the evil arises, which takes place in constipation; for when the colon is much distended with hardened fæces, it must press with some force on the ascending part of the duodenum, thus still further impeding the progress of the contents of the latter which have also to rise against gravity, when the body is in an erect position or recumbent on the right side. For this reason, it is always adviseable, previous to giving an opiate glyster where there is violent pain and vomiting from a gall-stone sticking in the duct, especially if the bowels have not been recently evacuated, to direct an opening enema to be injected, to unload the colon of those contents which, by distending it, cause pressure on that very part of the duodenum where the ductus communis opens, and thus impede the passage of the stone into the intestine. Be it recollected too that this bend of the duodenum is exactly between the right kidney and the ascending part of the colon, and that therefore much distention of it would in fact force the duodenum against the hard substance of the kidney, and would contribute to obliterate its cavity by the pressure of its sides against each other." 330.

The duodenum is very peculiarly and securely situated. It is studded with glands, extremely vascular, and its nerves have an extensive communication with the rest of the body by connexion with the par vagum and great sympathetic, through the medium of the semi-lunar ganglia.

"It is the most important recipient in the human body, receiving more various kinds of ingesta than any other organ: and notwithstanding it is less than the stomach, it has a greater capacity than the other intestines except the colon. It was necessary that the stomach should not only be greater because it receives the food in the gross, if I may so express it, but on account of the large quantity of ingesta, particularly of liquids which is sometimes thrown suddenly into it. The duodenum on the contrary receives the food gradually through the pylorus, and in a small quantity at a time, and in a more homogeneous state." 335.

In a pathological point of view, it is evident, as Dr. Yeats observes, that the duodenum is as liable to all the consequences of imperfect digestion as the stomach, with the probability of their being more distressing in the duodenal affection, in consequence of the elaborate manner in which this intestine is constructed, and the important fluids poured into it. It is probable, Dr. Yeats thinks, that the duodenum may have considerable power to correct imperfections in the gastric digestion; but if imperfect digestion take place in the duodenum, it does not seem likely, from their structure or functions, that any improvement can occur in subsequent portions of the alimentary canal.

The extrication (probably *secretion*) of gas is a very constant attendant on indigestion of our food. And from the situation and connexion of the duodenum, the distention this occasions must be productive of various distressing effects, both in the neighbourhood of the organ, and at great distances.

"The duodenal nerves come off from the right hepatic plexus, which also supplies the gall-bladder, biliary ducts, pylorus, pancreas and kidneys, and then they pass on to the substance of the liver; hence we see that duodenal distension will, by nervous connexion, (independent of mechanical effects) produce irritation in all these parts. The intestines have their healthy peristaltic action maintained by the nervous influence from the solar plexus, through the nerves distributed to their fine and beautiful vascular coat; an irritation of these nerves will not only canse spasm, but, by continuance, inflammatory action. The duodenum is extremely liable to the same effects: it is supplied with nerves directly from the eighth pair which communicate with the stomach and pylorus; thus besides the universal connexion between the par vagum and sympathetic, it receives some filaments from the semi-lunar ganglions and hepatic plexus; and as at its lower end it receives nerves from the superior mesenteric plexus which communicates with the renal plexus, it is clear how a morbidly irritated duodenum will produce uneasiness in nearly the whole of the abdominal viscera, causing irritation in the intestines, stomach, œsophagus, kidneys, the right particularly, liver, &c. &c. and as the hepatic plexus is formed from the semi-lunar ganglions, in which are concentrated not only the nerves which supply the chylopoietic viscera, but in which terminate those nerves which, in their progress from the brain, either give branches to, or inosculate with, nerves which supply the œsophagus, lungs, heart, diaphragm trachæa, larynx, pharynx, chest, muscles of the face, lower jaw, and neck; hence the headach, vertigo, contortions of the countenance, rolling of the eyes, asthma, cough, pains about the chest and shoulders, which accompany duodenal irritation. And that while oily emulsions and expectorants, which only add to the mischief, have been prescribed in such coughs and asthmas, medicines of a very different class should be given." 340.

Dr. Yeats might have quoted Hoffman in support of the above sentiments. Speaking of morbid biliary secretions in the duodenum, Hoffman remarks—" tenerrimas membranas duodeni flatibus distentas corripit, quæ dein in consensum trahunt diaphragma, plexus mesentericos stomachales, pneumonicos, et sic violenta fit tussis, quandoque cum vomitu et suffocationis metu, &c. ***** Quandoque etiam affectus istorum succorum in duodeno primisque intestinis stagnantium usque ad caput se exerunt, et cephalalgias, vertiginem, torporem omnium sensium, imo apoplecticos insultus ibi machinantur."*

Here Hoffman relates some curious cases, and quotes others, illustrative of the various sympathetic affections of distant organs and parts, produced by irritating secretions in the primæ viæ—facts which appear *new* to some physicians of the present day, who do not look back a little among the works of the illustrious dead.

Dr. Yeats goes on to observe, that in addition to these distant symptoms produced by nervous sympathy, painful sensations of a more local nature, arising from mechanical pressure of the distended duodenum, will take place. Pain and spasm will then be produced; and pressure on the biliary and pancreatic ducts will obstruct the egress of their respective secretions, giving rise to soreness and sense of fulness at the pit of the stomach, and a jaundiced appearance of the skin. Under these circumstances, Dr. Yeats has no doubt, (indeed we know many instances of it) that patients have been salivated for disease of the liver, when this last organ was perfectly sound in structure, and only disordered in function, through consent with the duodenum.

---

* De Duodeno, tom. iii. p. 192, fol. ed. *Genev.* 1761.

" Should it so happen, that calculi are contained in the gall-bladder, the pressure from the distended duodenum will cause additional pain by rubbing the rough stones against its sides. A great deal of pain will be felt across the back, and especially in the region of the right kidney, from the connexion which the duodenum has with its capsule, and a quantity of pale urine is often past, from irritation of the renal vessels, both locally and through the medium of the renal plexus of nerves." 343.

Dr. Yeats thinks, and not without reason, that all this distention and pressure must, more or less, obstruct the circulation of the bowel itself, and thus keep up an inflammatory irritation there. The portal circulation will also be impeded, and thus, plethora produced in the meseraic veins, with a varicose state of vessels in the legs, or hæmorrhoidal affections. These circumstances have been very pointedly alluded to by Hoffman.

" Videlicet dum intestinum vehementer distenditur, non modo tunicæ, quæ exquisito sensu pollent, affliguntur, sed et rami plexus mesenterici tenduntur; nec non, *vasis sanguineis compressis*, congestio sanguinis fit circa truncum venæ portæ, et initium arteriæ meseraicæ: sequitur hinc fixus in lumborum prima vertebra dolor, præcordiorum anxietas, appetitus dejectio, somni et virium defectus, alvique adstrictio." *Hoffmani Opera, Tom.* IV. *p.* 191.

That accurate anatomist, and able pathologist, Mr. Charles Bell, observes thus :

" We find that there are poured into the duodenum from the liver and pancreas, secretions which have an extensive effect on the system of the viscera; and we must acknowledge, that the derangement of these secretions must operate as a very frequent and powerful cause of uneasiness, and that the duodenum must often be the seat of disease and distressing symptoms. We may observe that, from the course of the duodenum, pain in it should be felt under the seventh or eighth rib, passing deep, seeming to be in the seat of the gall-bladder, and stretching towards the right hypochondrium, and to the kidney, and again appearing as if in the loins." *Vol.* III. *p.* 281.

We have seen two distinctly marked cases of duodenal disease, where pain in the *loins* was the most distressing symptom of all. Dr. Yeats next observes, that the duodenal affection soon leads to unhealthy discharges from the liver and pancreas, causing a great accumulation of irritating matter in the sacculated portion of the duodenum, with torpor throughout the whole line of the alimentary canal, and languor and lassitude of body. Dr. Yeats thinks, that the confinement of the duodenum at its termination within the ring at the root of the mesentery, will, also, increase the difficulty in the propulsion of the duodenal contents: consequently, at this point, the more indigestible and fœcal portion of the food, may sometimes lodge, and produce a temporary obstruction of the passage. Our author has no doubt that, repeated absorption by the lacteals of the acrid contents of the duodenum, produces disease and enlargement of the mesenteric glands, and ultimately tabes mesenterica.

"The liver, pancreas, and duodenal glands, will become diseased from congestion and irritation, and this irritation will be propagated by nervous communication to the brain; it will, sooner or later, run into inflammatory action and produce effusion there, or the irritation will chronically continue giving rise to spasms, convulsions, vomiting, contortions of the countenance, affections of the sight, violent headachs, faultering voice, chorea and palsy." 350.

The following symptoms in adults have been caused by duodenal disease, and have been mistaken for a liver complaint, to our author's knowledge. The appetite declines, or becomes capricious; flatulence is troublesome; languor and lassitude are considerable, accompanied by sense of weakness in the lower extremities, occasional chills, feverish heats, pain and sense of weight in the right hypochondrium and across the loins, torpid bowels with dark coloured fæces passed without being figured; urine of a mahogany colour, sometimes transparent, sometimes with a lateritious or white sediment, or thick like gruel and water; slight nausea; restless nights; white crusted tongue, brown towards the roots; occasional giddiness or headache, or affection of vision; pulse little accelerated—sometimes preternaturally slow, or intermitting—train of nervous symptoms. The food causes oppression—a dyspnœa, with, sometimes, a troublesome cough, adds to the restlessness of the nights—despondency, and, after some time, a yellow tinge in the eye, take place.

"Upon examining patients with the above symptoms, a fulness and puffiness will be sometimes perceived to the right of the pit of the stomach. I say sometimes, because it will depend on the quantity of extricated gas confined in the duodenum, or upon the existence of morbid action at the time, whether this symptom be present or not. By pressure *on* the region of the liver no uneasiness will be complained of, but if the pressure be made with the edge of the open hand under the ribs with the palm of it flat upon the abdomen, considerable uneasiness will be complained of up towards the liver and down towards the right kidney, a soreness too is felt an inch or two to the right just above the navel. Such patients will trace with most anatomical accuracy, the course of the duodenum with their finger, from the stomach to the loins on the right side, and back again across the abdomen to the umbilicus." 353-4.

Under such circumstances, our intelligent author observes, that the general routine of giving a few doses of calomel and salts, and then stimulants and tonics, produces no permanent good effects, "for the duodenum relapses into its inactivity after a brisk purge," particularly of calomel. The stimulus of ammoniacal salts gives only temporary relief, and should spirituous liquors be resorted to, they increase the evil, and induce organic disease. "The preparations of steel create nausea; the simple bitter infusions lie cold and heavy on the stomach, and the addition of aromatic or spirituous stimulants do not remove the difficulty."

"Notwithstanding, however, that bitters disagree, yet, when combined with a cathartic in such quantity as to give a gradual evacuating movement to the intestinal canal, they produce the happiest effects, ℈j. of quassia, and the same quantity, or ℈ij. or ʒi. rarely the last, of senna infused in a pint of boiling water for an hour, produce the composition I employ; f℥iss. of this infusion taken in the morning and repeated at noon, with pil. hydrarg. gr. iij. every night for about a fortnight, most generally remove this uneasy state of the duodenum." 357.

Hoffman has given nearly similar counsel in these cases. "Itaque ubi ejusmodi casus incidit, non satis est, dictis remediis ægrotantem tractare, sed validiora adhibere oportet, quem in censum referenda *aperientia fortiora, salia, et quæ ex Rhabarbaro, et mercurio dulci* constant medicamenta."

We have taken several occasions, in this Journal, to hint that, practitioners of the present day appear almost to have discarded *emetics* from their prescriptions, and depend entirely on purgatives. Yet we have seen such decidedly good effects from emetics, judiciously administered, that we cannot help recalling the attention of the profession to this almost exploded remedy. In the class of complaints under review, gentle emetics are often serviceable, and we recommend our brethren to reflect on the following sentence of the experienced Hoffman.

"Quod praxin et viam medendi attinet morborum, qui in duodeno et prima regione sedem fixerunt, facile ex dictis apparet; emetica diligenter præparata et prudenter exhibita hisce morbis unice auxiliari. Emetica sunt generosa et valida remedia." *Opera, Tom.* III. *p.* 195.

Although somewhat out of place, yet we cannot help here observing, for the gratification of the Broussaian School, that Hoffman joins his testimony to that of Helmont, of Sylvius, and of Riverius, that the real seat of *fever* is in the primæ viæ—" nidus febrium est in *primis officinis*, extenditur scilicet à pyloro ad duodenum, &c." *Loco citato.* This is precisely the *new doctrine,* about which our continental brethren are so fiercely contending. We fear, however, that neither its novelty nor antiquity will enable it to do more, than "strut and fret its hour upon the stage," and then die away, perhaps to be again and again resuscitated in future ages—

————— ut unda impellitur unda,
Urgeturque prior veniente, urgetque priorem.

When this morbid condition of the duodenum is attended with feverish heats, and high-coloured scanty urine, our author adds to the bitter, instead of the senna, some neutral salt, particularly the sulphat of soda, which, he thinks, has a more specific effect on the duodenum, than the sulphat of magnesia. He gives a scruple twice a day in the quassia infusion, and three grains of the pil. hyd. with or without aloes, according to the state of the bowels. When symptoms of inflammatory action prevail in the duodenum, the saline draught, with sulphat of potash, and the blue pill alone, are highly useful. A proper attention to diet and exercise is necessary of course.

"It is here a matter of more than medical curiosity, to form a discrimination between a liver disease on one hand, and a simple dyspeptic state of the stomach on the other, as contra-distinguished from a morbid state of the duodenum simply. In some cases where the duodenal disease has existed for a considerable time, the diagnosis becomes almost impossible, although an experienced practitioner may form a very good opinion; because a morbid train of symptoms takes place, by which liver, duodenum, and stomach, are blended in one affection, emphatically termed, a disease of the digestive organs. In a simple disease of the stomach, we have very little swelling or puffiness in the epigastric region, and when it does take place, it is more to the left side. We have also, eructations of wind and of acrid matters. These circumstances vary very considerably in a diseased duodenum; a swelling is very apt to occur, the extricated gas finding a greater difficulty to escape, either by regurgitation through the pyloric orifice, or downwards, from the particular situation of the intestine at the mesenteric ring. There are, therefore, no eructations of wind or of acrid matters in a dyspeptic state of the duodenum; and when the puffiness is detected, it is a diffused swelling towards the right hypochondrium, being lost under the liver, and not extending to the left side, and circumscribed in that direction.

"A diagnosis between affections of this intestine and the liver is more difficult, on account of the latter being so readily affected by a disease of the former, particularly when a slight jaundice has taken place, not from a diseased liver, but from the bile not finding a ready passage into the ill-conditioned duodenum; an experienced physician must then form his opinion from the accumulation of symptoms, and from his recollection of similar cases. If the symptoms of a liver disease, particularly the yellowness of the eyes, tension of the side, and lateritious sediment of the urine, speedily disappear by the treatment, we may be perfectly satisfied that these hepatic symptoms are produced by duodenal irritation, and that the patient may be safely tranquillized on that ground by his anxious physician; if on the contrary, these symptoms are obstinate, and they seldom are without having so much disordered the general system as to awaken, very early, the suspicions of the physician, then that science and accumulation of facts, which taught him the discrimination, will immediately suggest to him the more active and appropriate remedies for the liver disease according to its kind and degree." 361-2-3,

But our limits are exceeded, and we must now close the analysis of a paper which does great credit to the acute observation of its very able and ingenious author. Two plates and some pages of explanation, conclude this interesting article.

XIV. *Observations on the Hour-glass Contraction of the Uterus.* By John C. Douglas, M.D. Licentiate of the College of Physicians, Ireland.

This is a very sensible paper, and we are convinced of the truth of the doctrine there inculcated, from many observations made in the early part of our obstetric practice, when we were in the habit of meeting—or rather *producing*, what are termed hour-glass contractions of the uterus, by officiously and awkwardly groping about in the dilated vagina, before the uterus had time to contract and detrude the placental mass.

Authors have generally attributed retention of the placenta to hour-glass contraction, morbid adhesion, or inaction of the uterus. Dr. Douglas adduces it as his opinion, that the placenta is rarely, if ever, *primarily* retained by the kind of uterine contraction under consideration—" its formation (hour-glass contraction) being merely the result of the undecided manner in which the practitioner introduces, or attempts to introduce, his hand, with the intent to extract a placenta that had been retained by one of the other two causes."

Instead of the usual division of the uterus into fundus, body, and cervix, without any positive limits, our author would dispose of them thus:

" I would so arrange them as to apportion $\frac{2}{7}$ to its fundus, $\frac{3}{7}$ to its body, and $\frac{2}{7}$ to the cervix. By this division, the upper and lower sections on the long diameter of the uterus, are of equal length; and the middle section is equal to one and a half of either." 386.

But as the uterus and vagina, at the period of delivery, form one continuous canal, our author would divide the combined two, into four portions, and of the entire, assign $\frac{2}{10}$ to the fundus, $\frac{3}{10}$ to the body, $\frac{2}{10}$ to the cervix uteri, and $\frac{3}{10}$ to the vagina. In this arrangement we find the fundus and body to comprise the entire of the thick muscular substance of the womb, while the portion allotted to the cervix, partakes more nearly of the structure of the vagina. Instead, therefore, of considering, at the period of labour, the entire of the uterus as one organ, and the vagina as another, it would be more proper, Dr. D. thinks, to consider the fundus and body as one, possessing a common structure, and the cervix uteri and vagina as another, being, also, of a nearly common texture. The physiology of the parts countenances this arrangement. The functions of the cervix uteri, during labour, are the very reverse of those of the fundus and body. These last, exert a contractile and expulsive action, while the cervix relaxes in sympathy with the vagina. Our author remarks, en passant, that, in his experience, the protraction of labours has arisen more frequently, from "want of yielding elasticity in the cervix uteri and vagina, than from deficiency of expulsive action in the body and fundus." Here Dr. Douglas asks the practitioner if, in any case, after having overcome, what is termed, the hour-glass contraction, he has ever found the placenta in the upper chamber detached? —We can only answer for ourselves, that we never did. And we can well remember the painful stricture on our arm, while slowly and carefully detaching the placenta from the fundus uteri. If general experience shews (and our author thinks it will) that the adhesion, in question, always obtains, it will doubtless go far to prove, that the detention of the after-birth is owing to this, rather than to the contraction of the uterus. To account for this contraction, Dr. Douglas observes, that the practitioner, while searching in vain for the placenta, unconsciously irritates the lower edge of the thickly muscular part of the uterus into action:—

"The hour-glass contraction is the usual result of this irritation; and, by the time the practitioner has discovered his error, a barrier is thus opposed to the further progress of his hand." 391.

Sometime after adopting the above *theory*, our author seized an opportunity, in a case of retention, to introduce his hand just within the cervix uteri, delaying it there designedly until "the uterine cavity gradually assumed the hour-glass form." He then quickly passed up his hand to the fundus, without allowing the constriction to close so much as to materially impede its progress.

It had always appeared to our author an enigma that this hour-glass contraction, as it is called, should be inherent in a circular band of fibres at the centre of the uterus, and that the same structure, both above and below this belt, should remain quiescent and relaxed.

"But this stricture does not form from the middle circumference of the uterus; it is formed by the lowest verge of its thickly muscular substance at the line of demarcation of its body and cervix. Which line, in my arrangement of parts, is at an equal distance from the os externum of the vagina and the farthest part of the fundus uteri." 393.

From this it would appear that the upper chamber comprises the entire of the body and fundus, while the lower engages only the cervix and vagina, the two compartments being nearly equal in capacity. We shall sum up our author's conclusions in his own words, viz.

"That the remote cause of the uterus assuming the hour-glass form, is a miscalculation of the distance (which is not less than fifteen inches) at this period, from the os externum to the fundus of the uterus.

"That the exciting cause is irritation, produced either in the vagina, by injudicious pulling at the umbilical cord; or, in the cervix uteri, by the accoucheur's hand searching there in vain for the placenta.

"That the proximate cause is a spasmodic constriction of the muscular fibres of the uterus at the lower verge (not at the centre) of that section termed its body, and just where it ceases to be thickly muscular.

"Thence, I conclude that this hour-glass contraction is not produced by any principle of action inherent in the uterus itself; and that whenever it does occur, it is caused by mismanagement.

"Therefore, in order to avoid such occurrences, the practitioner should always refrain from exciting unnecessary irritation.

"And, in those few cases of unavoidable retention of the placenta, wherein it may be necessary for the accoucheur materially to interfere, he should, having first cautiously inserted it within the vagina, push his hand briskly up to the very fundus of the uterus. And, in this operation, he should direct the hand forward towards the umbilicus; ever bearing in recollection that the axis of the uterus, as well as the axis of the pelvis, inclines at a considerable angle to the axis of the trunk of the body." 397.

Our author's reasonings appear to us not only ingenious but solid; while the practical indications which result from them are useful and safe to act upon.

XV. *On the Necessity of Caution in the Estimation of Symptoms in the last Stage of some Diseases.* By Sir HENRY HALFORD, Bart. M. D. F.R.S. Now President of the Royal College of Physicians.

PROGNOSIS is by far the most difficult branch of medical science. To distinguish one disease from another, or to treat the complaint when known, is comparatively easy; but to say how a disorder will terminate, especially when it has advanced far, and already menaced life, requires the judgment of a master—of a man long conversant with the vicissitudes of disease, the extent of Nature's power, and the resources of human art. That old age too often generates a degree of caution bordering on timidity, in the physician, is not to be denied, but to be regretted as one of those inevitable failings of our nature that mark the hand of Time desolating the mansion of Thought, and enfeebling the energies of the soul. There is, however, another species of caution, not seldom confounded with the foregoing by the precipitancy of youth, the temerity of inexperience, or the arrogance of ignorance. It is that prudential reserve in delivering a prognosis which is grounded on a knowledge of the uncertainty of events, and the fallacy of appearances, acquired by long and careful habits of reflection, amid scenes of sickness and death. This is the species of caution which is recommended to our notice by a man who has arrived at that period of life when the judgment is matured by experience, but unimpaired by years. It is a caution, from non-attention to which we have often felt the mortification arising from false predictions—and the misery of having inspired hopes when ruin was impending, and despair at hand. We presume then, that every man who has any regard for his professional character and moral feelings will ponder with us on the following observations which Sir Henry Halford has offered to his medical brethren.

Our author truly remarks, that it is of great importance to be able to foretel the issue of a disease. When it is of a fatal nature, and this is not stated to the friends, their grief is rendered doubly poignant by the idea that the physician himself was taken by surprize, and therefore probably had not made use of all the resources of his art, by which the catastrophe might possibly have been prevented. On the other hand, a prudent and feeling disclosure of his apprehensions would have mitigated the sorrow of the relatives, whose conclusion would be that every thing which skill could suggest had been done to avert the hand of death. Sir Henry Halford properly observes, that the art of physic is so far conjectural, as all reasoning must be, which presumes on what *will* happen from what *has* happened—the only legitimate reasoning of which the science of medicine, in common with many other sciences, admits.

"And it suggests therefore, the necessity of recording facts, carefully ascertained by repeated experience. Were this done by every physician of extensive practice, what appears extraordinary in a single instance, would become familiar by repeated observation, and the difficulty of prognosticating would be materially diminished; to the great credit of physic, and to the satisfaction of its professors." 400.

Our author remarks that, towards the close of some disorders, both acute and chronic, especially where the constitution has had a violent and protracted struggle with the disease, appearances present themselves of a very equivocal and delusive nature, with which the issue of the malady does not correspond. Here Nature appears to pause for a moment, after the disease has done its worst—the frame is exhausted by its own efforts, and a general tranquillity pervades the whole system. The eager wishes of friends are apt to misconstrue this condition into the commencement of recovery, especially as the patient generally admits that he is better, having lost some of his sufferings. The physician here must be cautious how he indulges the same lively hopes, lest he compromise his character, and aggravate the feelings of the family.

Sir Henry has seen this fallacious truce in four or five instances of phrenitis. We shall give the following case in the words of our able and experienced author.

"A young gentleman of family, about twenty-five years of age, took cold whilst under the influence of mercury. The fever increased daily until it was accompanied at last by so much excitement and delirium, as made it necessary to use not only the most powerful medicines, but also personal restraint. At length, after three days of incessant exertion, during which he never slept for an instant, he ceased to rave, and was calm and collected. His perception of external objects became correct, and they no longer distressed him, and he asked, pressingly, if it were possible that he could live? On being answered tenderly, but not in a way calculated to deceive, that it was probable he might not, he* dictated most affec-

* "My friend Dr. Heberden, when I mentioned this case to him, shewed me a note which his father had received from a patient, written in the interval of the subsidence of a paroxysm of phrenzy and his death, which happened about fifteen hours afterwards. The note is of some length, and is written correctly.

See the chapter of Aretæus on the Καυσος, as remarkable for the sublimity of the ideas which it contains, as for the beauty of the Ionic Greek in which they are expressed."

tionate communications to his parents abroad, recollected some claims upon his purse, "set his house in order," and died the following night. The reason why so unfavourable an opinion was entertained of his state, was, that the apparent amendment was not preceded by sleep, and was not accompanied by a slower pulse, two indispensable conditions on which only a notion of real improvement could be justified. But here was merely a cessation of excitement occasioned by a diminution of power, and by a mitigated influence of the action of the heart upon the brain." 404.

It is notorious, as Sir Henry Halford observes, that, in inflammation of the bowels, mortification often follows a cessation of pain; but in that partial inflammation produced by a strangulation of a portion of bowel in hernia, how often have we occasion to deplore the broken hopes of relatives after a surgical operation had promised that all was well. It is an invariable, and we think very prudent, rule with our author, still to consider life in jeopardy until the intestines again perform their functions, the stomach is free from irritation, and the skin remains universally and agreeably warm.

An abscess in the liver, connected with gall-stone, sometimes assumes the type and character of an intermittent fever, and may be incautiously mistaken for, and treated as, this last affection. The preceding hepatic inflammation—the violence of reaction in the second stage—the affection of the brain, amounting to an apoplectic stupefaction—the deep brown tinge of the skin in the paroxysm—these will enable the attentive physician to discriminate the periodical accessions of fever, in hepatic abscess, where they occur, from common miasmal intermittent. Our experienced author saw three instances of this masked disease, two of whom died in the fourth paroxysm—the life of the third was protracted a fortnight, in consequence of the abscess bursting into the channel of the intestines, and the matter passing off in large quantities by stool.

In hydrothorax Sir Henry Halford cautions the physician to be on his guard, when delivering an opinion in the advanced stages of the disease. A material mitigation of the dyspnœa generally takes place when the legs swell, and may thus induce the medical attendant, as well as the patient and friends, to entertain too sanguine hopes that the hydropic affection of the extremities is the only remaining malady to combat. Our author remarks, "that if this swelling of the legs disappear without an increased discharge of urine, the patient generally dies very soon, and most frequently suddenly. Whereas, if an ample increased secretion by the kidneys *follow* the relief of the dyspnœa, then every good hope of a temporary recovery, at least, may fairly be entertained, though it should be acknowledged that this species of dropsy, above all others, is most apt to return."

The confluent small pox, in a certain stage, requires, our author observes, a guarded prognosis.

"The physician may fairly acquiesce in the fears of a family, when, on the completion of the eruption, he sees the face and breast one mass of disease, and may most reasonably doubt the capability of the constitution to maturate and perfect so large an eruption. But he must not hold out unfounded hopes to the parents if the malady proceed in the next stage, in a most satisfactory manner, beyond his expectations;—the pustules ripening fully, and the process being complete—for alas! at this very moment it may be, the patient is sinking—is dead! the powers of his constitution being exhausted by the efforts it has made, and no longer equal to the accomplishment of a protracted cure." 408.

Analogous, in some measure, to the maturation of variola, is the reparation of skin when extensively destroyed by fire. Our author has seen several instances of this kind, four of which proved fatal, "and yet, in every one of the four, the wound had healed, with the exception of the space only of a crown piece." Two of these died, "no warning given" by any particular symptom of danger. Prudence, therefore, will induce the practitioner to consider the patient in some danger, after extensive burns, till the wound has been entirely healed for some time, and the constitution has recovered its usual energy.

Of paralysis of the kidney, Sir Henry Halford has only seen five instances in a practice of twenty-seven years. The last happened about two years ago, and as it was an exact copy of all the others, we shall introduce it in the author's own words.

"A very corpulent robust farmer, of about fifty-five years of age, was seized with a rigor, which induced him to send for his apothecary. He had not made water, it appeared, for twenty-four hours; but there was no pain, no sense of weight in the loins, no distension in any part of the abdomen, and therefore no alarm was taken till the following morning, when it was thought proper to ascertain whether there was any water in the bladder, by the introduction of the catheter; and none was found. I was then called, and another inquiry was made, some few hours afterwards, by one of the most experienced surgeons in London, whether the bladder contained any urine or not, when it appeared clearly that there was none. The patient sat up in bed and conversed as usual, complaining of some nausea, but of nothing material in his own view; and I remember that his friends expressed their surprize that so much importance should be attached to so little apparent illness. The patient's pulse was somewhat slower than usual, and sometimes he was heavy and oppressed.

"I ventured to state that if we should not succeed in making the kidneys act, the patient would soon become comatose, and would probably die the following night; for this was the course of the malady in every other instance which I had seen. It happened so; he died in thirty hours after this, in a state of stupefaction." 412.

All the patients of this kind were fat corpulent men, between fifty and sixty years of age; and in three of them "there was observed a remarkably strong urinous smell in the perspiration twenty-four hours before death." The smallest quantity of urinary secretion, in such cases, should inspire hope, Sir Henry thinks, since it is surprizing how trifling a measure of that excrementitious fluid is compatible with life and even health. "But the cessation of the excretion altogether, says our author, is universally a fatal symptom, in my experience, being followed by oppression on the brain."

Practitioners who are anxious to discharge their arduous duties, with honour to themselves, and satisfaction to their employers, will do well to bear in record these admonitory hints, (the fruit of long experience) from a man whose talents, erudition, and amenity, have conducted him with honour, and apparently without an enemy, to the enviable summit of his profession.

XVI. *Case of Death by Poison, wherein impregnation had taken place, and the Ovum was detained in the Ovary, with an Engraving.* By Edward Stanley, Assistant Surgeon, &c. &c. at Bartholomew's Hospital.

This is an interesting account of an extra-uterine fœtation by that accurate and zealous young surgeon and anatomist, Mr. Stanley. The unfortunate female had put an end to her existence by laudanum, and the general appearances, *post mortem,* are minutely detailed by our author. These we shall pass over, and come to the uterine system. The uterus itself was larger than usual, its substance being soft, and its internal surface covered by a layer of a yellowish white colour, of soft and spongy texture; exhibiting the usual characters of the decidua. The fallopian tubes were enlarged and remarkably twisted. The left ovary was larger than the right, and at its posterior part was a rounded prominence distinct from the general fulness. The external membrane of the ovary did not shew any appearance of aperture. On this being divided, a distinct cyst was exposed, and within the cyst an ovum. The chorion and amnios were perfectly distinct, the cavity of the latter being filled with a yellowish honey-like matter, but no fœtus could be found.

Mr. Stanley concludes with some important jurisprudential remarks on the proofs of impregnation, before the ovum can be distinctly seen. The existence of a corpus luteum is no longer held as conclusive evidence, since excitement of the ovaries, from passion or unfruitful connexion, is sufficient to rupture the vessels and produce corpora lutea. Neither is the decidua in the uterus proper evidence, since that is produced, as our readers know, in some cases of difficult menstruation. Our opinion must be formed from a review of all the circumstances appertaining to the condition of the uterus, ovaries, and fallopian tubes—always recollecting, however, that nothing short of the actual inspection of the ovum can be regarded as decisive testimony upon the subject.

---

We have now, we hope, presented to the profession a full and impartial analytical delineation of this volume, and thereby enabled the public to form a just estimate of its value as a whole. We are persuaded that our brethren at large will feel grateful to the contributors to the work, collectively and individually; and that, with us, they will hail with pleasure its annual or biennial returns, and deplore the slow pace of a quinquennial revolution.

